# Acute Finger Ischemia Evaluated With Point‐Of‐Care‐Ultrasound and Diagnosed With Concurrent Polycythemia Vera, Antiphospholipid Syndrome, and Tobacco Use

**DOI:** 10.1002/ccr3.72886

**Published:** 2026-06-08

**Authors:** Tristan Burgess, Michael C. Larkins, Titus Chu

**Affiliations:** ^1^ Department of Emergency Medicine, Boonshoft School of Medicine Wright State University Fairborn Ohio USA

**Keywords:** antiphospholipid syndrome, color doppler, finger ischemia, hyperviscosity syndrome, point‐of‐care ultrasound, polycythemia Vera, vascular ultrasound

## Abstract

In patients with undifferentiated extremity ischemia, point‐of‐care ultrasound (POCUS) can be used for rapid, non‐invasive assessment and diagnosis. POCUS can be used to assess for both arterial and venous occlusion and localize the extent of ischemia. Important considerations in such patients include polycythemia vera and antiphospholipid syndrome, and while rare, multiple coagulation defects can be present concurrently.

## Graphical abstract

We present a case of digital ischemia investigated and localized using point‐of‐care ultrasound (POCUS), in a patient that was subsrquently diagnosed with antiphospholipid syndrome and polycythemia vera concurrently, in the setting of active tobacco use.

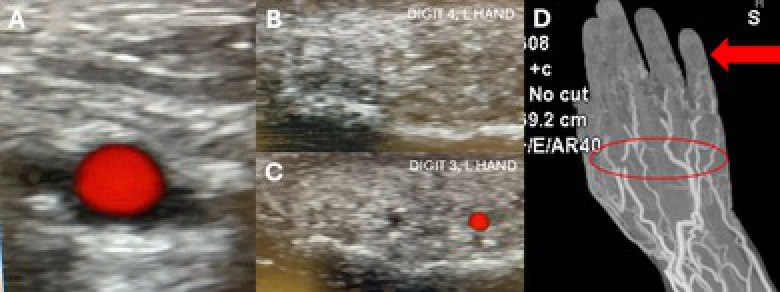

## 1 Introduction

Critical digital ischemia is a rare subset of acute limb ischemia often resulting in functional impairment or tissue loss [1, 2]. Etiologies include hypercoagulable states, atrial fibrillation, thromboangiitis obliterans, vasospasm, trauma, and neurovascular compression in the proximal limb [3]. The incidence of digital ischemia itself is not well documented, though estimates exist of around two cases per 100,000 per year [4]. Specific numbers regarding the incidence from hypercoagulable disorders is not currently known.

Polycythemia vera (PV) is one of three myeloid malignancies known as myeloproliferative neoplasms and is characterized by erythrocytosis, often with leukocytosis and thrombocytosis [5]. It is associated with mutations of the Janus kinase (JAK) 2 protein. The incidence and prevalence of PV is 0.01 to 2.61 and 0.49 to 46.88 per 100,000, respectively. Antiphospholipid syndrome (APS), on the other hand, is a disorder characterized by autoantibodies that target phospholipid‐binding proteins [6]. APS has a reported incidence of 2.1 and prevalence of 50 per 100,000, respectively. Both disorders predispose patients to thromboembolism, and concurrent PV and APS predisposes individuals to an even greater risk of thromboembolism [7]. Furthermore, patients with JAK2 mutations have been found to have an increased incidence of APS, with those with a JAK2 mutation having a 22.4% incidence of concomitant APS compared to 8.4% among those without this mutation [7].

Point‐of‐care ultrasound has been used to diagnose vascular compromise in patients with limb ischemia, mainly regarding arterial occlusion or injury [8, 9, 10]. It has also been used to assess for deep venous thrombosis, and ultrasound scans of the upper extremities by ultrasound technicians are also commonplace in the Emergency Department (ED) [11]. Ultrasound has also demonstrated utility with the assessment of hand fractures and tendonous injuries [12], and has been reported as being useful in the evaluation of digital ischemia in the setting of Raynaud's disease [13].

While not explicitly reported in the literature, the use of POCUS to assess for the etiology of finger ischemia does have the potential to change management and drive ED disposition. This report demonstrates a case in which a patient presented with finger ischemia and POCUS was used to localize ischemic vasculature, while subsequent imaging, laboratory workup, and medical management with ED‐to‐ED transfer were carried out.

## 2 Case History/Examination

### 2.1 Patient Information

A 68‐year‐old female with a history of severe bilateral knee osteoarthritis (OA) and tobacco use presented to the ED after referral from her primary care provider for finger discoloration and pain in her left hand. She reported that her middle, ring, and pinky fingers of her left hand had spontaneously become dark purple at the tips, with progressively worsening pain not relieved by ibuprofen. She reported some associated numbness but could still use her hand to complete daily tasks, and reported that her pain, which was initially severe, had progressed to numbness a few hours ago. She denied recent trauma and reported chronic acetaminophen and ibuprofen use for OA but denied any use of aspirin, blood thinners, or other medications.

She reported her fingers were similarly discolored 1 week prior, following her smoking a few cigarettes, but resolved spontaneously. Patient reported she occasionally drank alcohol and smoked approximately 0.5 packs per day, with a total pack‐year history of 25. She denied a personal or family history of cancer or bleeding disorders.

### 2.2 Clinical Findings

**FIGURE 1:**
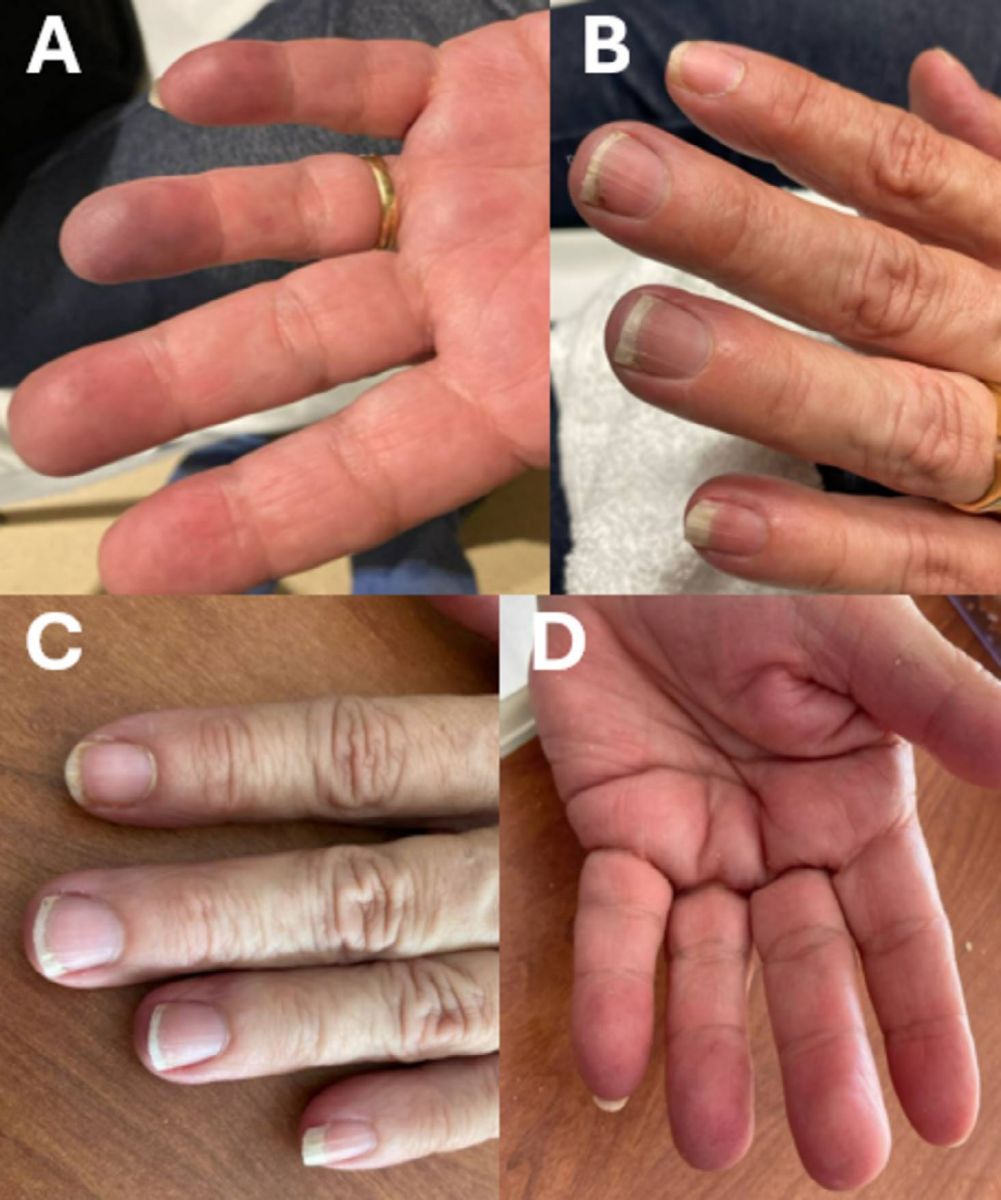
Finger discoloration of the left hand, digits 3–5, concerning for ischemia with subsequent improvement. The patient presented complaining of 24 h of worsening pain and discoloration to digits 3–5 of her left hand, which resolved to numbness a few hours prior to ED presentation. Pictures of her palmar (A) and dorsal (B) hand initially can be seen, as well as a splinter hemorrhage under the nail of digit 4 (B). Four days later, after treatment with heparin, dorsal (C) and palmar (D) pictures of the patient's left hand demonstrated improved discoloration with improved pain and numbness and resolution of the splinter hemorrhage (C).

Examination demonstrated mild purplish discoloration of the distal digits 3–5 (see Figure 1A,B), with numbness but full range of motion and normal grip strength. A splinter hemorrhage was noted under the nail of digit 3. The upper right extremity was unremarkable. Two+ radial and ulnar pulses were noted bilaterally, though prolonged capillary refill was noted in digits 3–5 on the left, worst in D4, which also had a gold wedding band in place without obvious swelling. Cardiopulmonary examination was unremarkable, with clear breath sounds bilaterally with regular heart rate and rhythm, without obvious murmur appreciated.

### 2.3 Differential Diagnosis, Investigations, and Treatment

#### 2.3.1 Diagnostic Assessment

**FIGURE 2:**
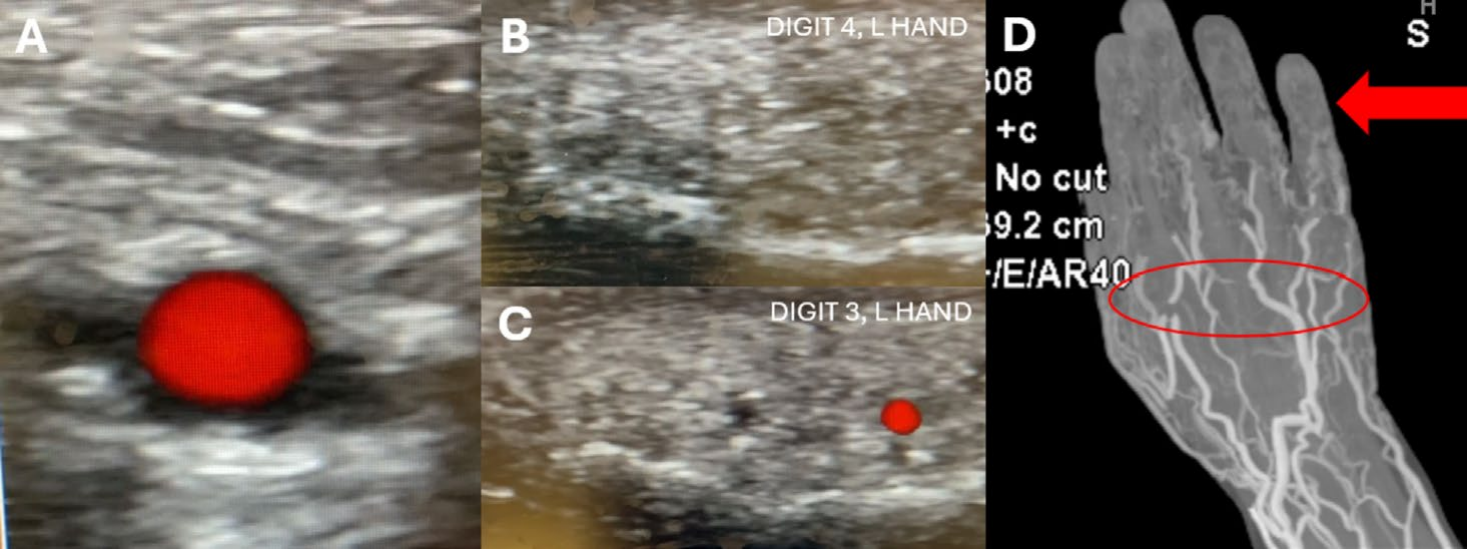
Point‐of‐care ultrasound (POCUS) and computed tomography angiography (CTA) imaging of a patient with finger discoloration and pain. POCUS imaging demonstrating strong color Doppler flow in the radial artery (A) and digit 3 (C) of the left hand. Digit 4, however, had absent color Doppler flow (B). Subsequent CTA (D) demonstrated an absent palmar arch (red circle) and hypoenhancement of the digital arteries (red arrow).

POCUS was utilized at bedside, which demonstrated patent radial and ulnar arteries in the left wrist with collapsible radial and ulnar veins. Color Doppler demonstrated appropriate flow in both systems (see Figure 2A). However, color Doppler assessment of the discolored digits in the left hand demonstrated absent flow in digits 4 and 5 without other obvious abnormality (see Figure 2B,C). Given the concern for occlusive pathology in the fingers, a heparin drip was initiated, the vascular surgery service was consulted, and computed tomography angiography (CTA) of the left upper extremity was ordered. Initial labs included a complete blood count, basic metabolic panel, prothrombin time, and activated partial thromboplastin time, which were notable for leukocytosis at 12.4 K/μL, erythrocytosis of 20.5 g/dL, and thrombocytosis at 694 K/μL. Table 1 demonstrates initial lab values for this patient. CTA demonstrated patent upper extremity arteries, with patent radial and ulnar arteries at the level of the wrist. However, no palmar arch artery was identified and hypoenhancement of the digital arteries was noted (see Figure 2D).

**TABLE 1:** Selected laboratory results for patient presenting with acute finger ischemia.

Laboratory test	Resultant value (units)	Reference range (units)
WBC count	**12.4 (K/μL)**	3.5–10.9 (K/μL)
RBC	8.08 (M/μL)	3.95–5.26 (M/μL)
Hemoglobin	**20.5 (g/dL)**	11.2–15.7 (g/dL)
Platelet count	**694 (K/μL)**	140–400 (K/μL)
Creatinine	1.0 (mg/dL)	0.5–1.2 (mg/dL)
Anti‐prothrombin IgG	1 (G unit)	< 20 (G units)
IgA, beta 2 glycoprotein	9 (A unit)	< 20 (A units)
IgG, beta 2 glycoprotein	3 (G units)	< 20 (G units)
IgM, beta 2 glycoprotein	**22 (M units)**	< 20 (M units)
Anticardiolipin, IgA	**35 (APL)**	< 22 (APL)
Anticardiolipin, IgG	**68 (GPL)**	< 23 (GPL)
Anticardiolipin, IgM	**13 (MPL)**	< 11 (MPL)
Phosphatidyl serine IgA	**22 (APS)**	< 20 (APS)
Phosphatidyl serine IgG	**55 (GPS)**	< 16 (GPS)
Phosphatidyl serine IgM	7 (MPS)	< 22 (MPS)
Erythropoietin	**2.3 (mIU/mL)**	2.6–18.5 mIU/mL
Rheumatoid factor	11.0 (IU/mL)	< 14 IU/mL
Uric acid	7.9 (mg/dL)	2.5–7.0 (mg/dL)
Sed rate	17 (mm/h)	0–30 (mm/h)
Anti‐nuclear antibody	< 1:40	< 1:40

*Note:* Bold denotes values outside of reference range.

Abbreviations: RBC = Red Blood Cell, WBC = White Blood Cell.

#### 2.3.2 Therapeutic Intervention

The patient was subsequently transferred following CTA and initiated on heparin treatment. The patient's weight was measured at 81.6 kg and she was given a loading dose of 4000 units of unfractionated heparin, followed by a heparin infusion dosed according to institutional algorithm based on anti‐Xa monitoring. She was admitted by the hospitalist service and evaluated by the vascular surgery service, who agreed with maintaining the heparin drip but did not see any benefit from surgical intervention. An arterial duplex scan was subsequently obtained, notable for dampened photoplethysmography waveform in the fourth digit of the left hand, concerning for decreased vascular flow. The patient was maintained on heparin for the next few days, and she was discharged on an aspirin 81 mg tablet once per day and transitioned on the day of discharge to rivaroxaban 15 mg tablets to be taken once per day for 3 weeks, followed by transition to one 20 mg tablet once per day.

#### 2.3.3 Follow‐Up and Outcomes

Given her erythrocytosis, the patient was also consulted to the hematology service, and subsequent genetic testing and rheumatologic labs were notable for a confirmed mutation of JAK2 V617F as well as elevated anticardiolipin IgA, IgG, and IgM (see Table 1). The patient was subsequently diagnosed with presumptive polycythemia vera and at this point presumptive APS. The hematology/oncology service recommended therapeutic phlebotomy at 500 mL daily until the patient's hematocrit resolved to < 45%–50%, which required a total of two liters of blood removed over the next 4 days, with a resultant hematocrit of 48%. At reevaluation 4 days after admission, the patient's finger ischemia had improved and the splinter hemorrhage noted previously had resolved (see Figure 1C,D). She was discharged in stable condition and remained on both rivaroxaban and aspirin 81 mg while following up with the hematology service outpatient.

Criteria for polycythemia vera were reviewed based on the World Health Organization 2016 diagnostic criteria [14]: given the patient's initial polycythemia (hgb 20.5 g/dL), subsequent JAK2 V617F mutation, and subnormal erythropoietin level (2.3 mIU/mL, with reference range 2.6–18.5 mIU/mL), the patient was conclusively diagnosed with polycythemia [14]. Additionally, there was strong consideration of antiphospholipid syndrome though the patient presented with possible small vessel occlusion (not large) and had multiple comorbid risk factors, including active tobacco use, obesity, and hypertension. The hematology/oncology team felt trialing the patient on rivaroxaban in place of warfarin, with simultaneous aspirin, was appropriate though off label.

Six days following discharge, the patient was seen outpatient in the hematology/oncology clinic for follow‐up. Complete blood count at this time demonstrated hematocrit of 49.4% and platelet count of 1044 K/μL, and thus an additional 500 mL of blood was drawn and wasted. The patient at this point was initiated on hydroxyurea 500 mg twice per day. One week later, the patient's blood was rechecked, and hematocrit was 48.7% and platelet count was 1135 K/μL. An additional 500 mL of blood was drawn and discarded, and the patient was increased to 1000 mg of hydroxyurea twice per day. One week later, the patient's blood was drawn again, with hematocrit now 47.7% and platelet count of 390 K/μL. She was decreased to 500 mg twice per day of hydroxyurea and remained on this dosing, stable, for the next 6 months.

Three months following admission, the patient underwent repeat testing for APS outpatient and was found to have persistently elevated anticardiolipin IgG (68 to 61 GPL), supporting a diagnosis of APS per the 2023 American College of Rheumatology (ACR)/European Alliance of Associations for Rheumatology (EULAR) antiphospholipid syndrome classification criteria [15]. See Table 2 for all values.

**TABLE 2:** Antiphospholipid testing for patient presenting with acute finger ischemia.

Laboratory test	Initial value (units)	Three‐month follow‐up value (units)	Reference range (units)
Anti‐prothrombin IgG	1 (G unit)	1 (G unit)	< 20 (G units)
IgA, beta 2 glycoprotein	9 (A unit)	6 (A unit)	< 20 (A units)
IgG, beta 2 glycoprotein	3 (G units)	1 (G units)	< 20 (G units)
IgM, beta 2 glycoprotein	**22 (M units)**	19 (M units)	< 20 (M units)
Anticardiolipin, IgA	**35 (APL)**	**30 (APL)**	< 22 (APL)
Anticardiolipin, IgG	**68 (GPL)**	**61 (GPL)**	< 23 (GPL)
Anticardiolipin, IgM	**13 (MPL)**	10 (MPL)	< 11 (MPL)
Phosphatidyl serine IgA	**22 (APS)**	19 (APS)	< 20 (APS)
Phosphatidyl serine IgG	**55 (GPS)**	**42 (GPS)**	< 16 (GPS)
Phosphatidyl serine IgM	7 (MPS)	5 (MPS)	< 22 (MPS)

*Note:* Bold denotes values outside of reference range.

At six‐month follow up, the patient reported intermittent dizziness and nausea thought to be secondary to her hydroxyurea and she was transitioned to 500 mg once per day. Her complete blood count at this time demonstrated a hematocrit of 40.4% and a platelet count of 568 K/μL. To date, she has not experienced any recurrence of thrombosis or been hospitalized again.

## 3 Discussion

Antiphospholipid syndrome (APS) is an autoimmune condition characterized by the presence of elevated antiphospholipid antibodies with subsequent clinical manifestations, generally thrombosis or pregnancy loss or complication [6]. The pathophysiology of this condition is not entirely understood, but a proposed two‐hit model involving endothelial injury followed by thrombus formation at the site of injury is widely accepted [16]. Polycythemia vera, on the other hand, occurs when cells in the bone marrow have a JAK2 kinase mutation resulting in signaling derangements and subsequent unregulated panmyelosis [5]. The generally accepted management of APS is treatment with warfarin among those with persistently high antibody titers, triple‐positive disease, or history of APS with arterial thrombosis; warfarin, while for PV management phlebotomy, aspirin, and lifestyle management are first‐line treatments, with cytoreductive therapy, usually hydroxyurea, reserved for high‐risk or refractory patients [16, 17].

While the exact incidence of concurrent PV and APS is not well known, it is generally agreed that patients developing both conditions is rare, though those with the JAK2 V617F mutation are likely at increased risk of developing APS [7]. It is agreed, though, that patients with both disorders are at even higher risk of thromboembolic events that those possessing just one such disorder. Such disorders were considered by the ED team during assessment, though definitive diagnosis of PV and APS is generally outside the realm of emergency medicine. Assessment of threats to life and limb is though, and in this case, POCUS was utilized to immediately assess and reassure against large vessel occlusion. It also allowed the localization of affected vessels and allowed the ED team to initiate heparin while discussing the case with the vascular surgery team and obtaining CTA imaging.

Per literature review there are no cases the authors could find regarding concomitant APS and PV causing finger ischemia, either venous or arterial in nature. Review of the literature demonstrated three case reports documenting complications in patients with both APS and PV: Bragagni et al. 2004, who describe Budd‐Chiari syndrome [18], Webber et al. who describe a hematoma found on a congenital bicuspid aortic valve [19], and Ameen and Pallivalappil who report a 25‐year‐old found to have splenic vein thrombosis with concomitant APS and PV [20].

Regarding diagnosis of APS and PV, the patient in this report was found to meet criteria for both on outpatient assessment and retesting for antiphospholipid antibodies. Persistently elevated anticardiolipin IgG > 40 GPL was seen at 12 week follow up. Regarding PV, while the patient did not undergo bone marrow testing, she did have an initial hemoglobin > 16.0, positive JAK2 V617F mutation, and subnormal erythropoietin level (2.3 mIU/mL, with reference range 2.6–18.5 mIU/mL), which meet the 2016 World Health Organization criteria for definitive diagnosis. It should be made clear that diagnosis and treatment of both diseases was presumptive until all criteria were met, which is not generally feasible in the ED.

We will note that the decision to treat the patient in this case with rivaroxaban and aspirin, rather than warfarin, contrasts with the current recommendation for patients with antiphospholipid syndrome experiencing arterial thrombosis to be treated on warfarin. The evidence has demonstrated such patients, or those that are so‐called triple positive (have elevations in all three primary APS antibodies, anticardiolipin, lupus anticoagulant, and anti‐beta2 glycoprotein), have increased incidence of repeat thrombosis on direct oral anticoagulants compared to warfarin [21]. However, the patient in this case did not have a definitive arterial thrombosis. It is highly likely she did, we admit, though if present it was small‐vessel. It was felt by the hematology/oncology team that had her thrombosis been large vessel, warfarin would have been indicated. Additionally, the patient had multiple comorbid conditions that could have been contributing to her isolated finger ischemia, including active tobacco use, hypertension, obesity, and uncontrolled PV. Furthermore, the patient preferred trialing aspirin and rivaroxaban along with lifestyle management prior to initiation of warfarin.

Another consideration was thromboangiitis obliterans (TAO), also known as Buerger Disease. Patient's present with pathognomonic finger or toe ischemia or ulceration while actively using tobacco [22]. While the exact etiology of TAO is not fully understood, current theories proposed involve immunologic dysfunction and tobacco hypersensitivity, leading to anti‐endothelial antibodies and impaired vasorelaxation. Definitive diagnosis would require biopsy, as no other laboratory testing is available to confirm this diagnosis and it is very much a disease of exclusion. Angiography has been reported demonstrating corkscrew arteries in affected limbs in patients with TAO, though these are not consistent in all patients and were not seen in the patient described in this case. Given the patient was diagnosed with two hypercoagulable conditions that would also lead to small‐vessel occlusion/hypoperfusion, the authors feel it is more likely the patient's condition was caused by PV and APS, though the patient was still counseled on tobacco cessation.

While ultrasound is routinely used in the ED for rapid assessment for life‐threatening conditions [23], there is limited literature on its use to assess for vascular flow in conditions outside of deep venous thrombosis or arterial occlusion/thromboembolism in the more proximal upper limbs [8, 9, 10]. Additional literature reports on the use of ultrasound, specifically duplex ultrasonography, in the evaluation of peripheral artery disease [24]. McMahan & Wigley do discuss the role of Doppler ultrasound in the assessment of blood flow in patients with potential Raynaud phenomenon, likely a similar process to what was affecting the patient we presented in this report [13]. However, we would like to emphasize that most literature on the use of ultrasound is centered on its execution by ultrasound technicians, often through the radiology department of a medical center. More recent literature has focused on the use of POCUS, particularly in the ED, perhaps by physicians or other staff, to assess patients quickly at bedside; there is literature that suggests that POCUS may be non‐inferior to conventional ultrasound in many ways and has the benefit of not requiring staff from another department to perform imaging [25].

In the case of our patient, POCUS allowed for instant assessment and localization of the affected region in the patient's hand. It gave valuable information, reassuring against other etiologies for the patient's finger discoloration and allowing the ED team to consult the vascular surgery service and arrange transfer more quickly. It is possible the time necessary for conventional ultrasound to be obtained may have resulted in a more prolonged hospital course and greater tissue loss, perhaps resulting in amputation.

Regarding patient perspectives, the patient expressed gratitude with her eventual diagnosis and improvement in symptoms. She reported discomfort with the phlebotomy as it was unsightly to have the blood pulled from her body but she appreciated it improving her headache. She also expressed frustration with having to take multiple new medications but was thankful to her healthcare team for her care.

In conclusion, digital ischemia is a rare presentation to the ED, and concurrent PV and APS are even rarer. Healthcare team members would benefit from the consideration of diverse etiologies for patient presentations. Treatment of these conditions involves anticoagulation and, in the case of PV, cytoreductive therapies and possibly phlebotomy. Definitive diagnosis of these conditions is an important component of patient care. Additionally, POCUS is a tool that allows for rapid assessment of patients at bedside and can be life‐ and limb‐saving when utilized competently. This case highlights the use of POCUS for assessment for vascular compromise and localization in patients presenting with concern for ischemia in the distal extremities.

## Author Contributions

**Michael C. Larkins:** conceptualization, data curation, formal analysis, funding acquisition, investigation, methodology, project administration, resources, software, validation, visualization, writing – original draft, writing – review and editing. **Tristan Burgess:** conceptualization, data curation, formal analysis, funding acquisition, investigation, methodology, project administration, resources, validation, visualization, writing – original draft, writing – review and editing. **Titus Chu:** project administration, supervision, validation, writing – original draft, writing – review and editing.

## Funding

The authors have nothing to report.

## Ethics Statement

The procedure described was in accordance with the institutional ethical guidelines and conformed to the WMA Declaration of Helsinki—ethical principles for medical research involving human subjects. Written informed consent was obtained from the patient for the publication of their medical case and any accompanying images.

## Conflicts of Interest

The authors declare no conflicts of interest.

## Data Availability

The data that support the findings of this study are available on request from the corresponding author. The data are not publicly available due to privacy or ethical restrictions.
